# Isolation, Characterization, and Transplantation of Cardiac Endothelial Cells

**DOI:** 10.1155/2013/359412

**Published:** 2013-10-27

**Authors:** Busadee Pratumvinit, Kanit Reesukumal, Kajohnkiart Janebodin, Nicholas Ieronimakis, Morayma Reyes

**Affiliations:** ^1^Department of Pathology, Institute for Stem Cell and Regenerative Medicine, School of Medicine, University of Washington, Seattle, WA 98109, USA; ^2^Department of Clinical Pathology, Faculty of Medicine Siriraj Hospital, Mahidol University, Bangkok 10700, Thailand; ^3^Department of Oral Biology, School of Dentistry, University of Washington, Seattle, WA 98195, USA; ^4^Department of Anatomy, Faculty of Dentistry, Mahidol University, Bangkok 10400, Thailand

## Abstract

Isolation and *ex vivo* expansion of cardiac endothelial cells have been a recurrent challenge due to difficulties in isolation, cell heterogeneity, lack of specific markers to identify myocardial endothelial cells, and inadequate conditions to maintain long-term cultures. Herein, we developed a method for isolation, characterization, and expansion of cardiac endothelial cells applicable to study endothelial cell biology and clinical applications such as neoangiogenesis. First, we dissociated the cells from murine heart by mechanical disaggregation and enzymatic digestion. Then, we used flow cytometry coupled with specific markers to isolate endothelial cells from murine hearts. CD45+ cells were gated out to eliminate the hematopoietic cells. CD31+/Sca-1+ cells were isolated as endothelial cells. Cells isolated from atrium grew faster than those from ventricle. Cardiac endothelial cells maintain endothelial cell function such as vascular tube formation and acetylated-LDL uptake *in vitro*. Finally, cardiac endothelial cells formed microvessels in dorsal matrigel plug and engrafted in cardiac microvessels following intravenous and intra-arterial injections. In conclusion, our multicolor flow cytometry method is an effective method to analyze and purify endothelial cells from murine heart, which in turn can be *ex vivo* expanded to study the biology of endothelial cells or for clinical applications such as therapeutic angiogenesis.

## 1. Introduction

Coronary heart disease is the leading cause of death in the United States, with more than 16 million people afflicted with this condition [1]. Treatments currently available include pharmacological therapy as well as revascularization therapy such as percutaneous coronary intervention and coronary artery bypass grafting to restore the blood flow to the compromised area of the heart [2]. Even with the available treatment, many patients remain symptomatic. Angiogenesis, the growth of new blood vessels, following an ischemic insult of the heart may help relieving symptoms and prolonging life expectancy. Therefore, understanding the behavior, nature, and response of cardiac endothelial cells (ECs) is instrumental for the development of future cardiac angiogenic therapeutics. Commercially available endothelial cell lines are widely used to study endothelial cell biology. However, endothelial cell lines may have lost important EC properties or functions. In addition, transforming agents used to immortalize these cell lines may affect cellular functions and impede their use for clinical applications [3]. Also, endothelial cell lines from only very few tissues are available. Mouse cardiac endothelial cell line has been described [4] by transfecting lentiviral vectors carrying SV40 T antigen and human telomerase. Random integration in the genome from lentiviral transfection may cause cancer *in vivo* and is not clinically applicable. 

EC are a heterogeneous population. This heterogeneity stems from differences in endothelial phenotype of different vessel type (arterial versus venous) and differences in EC phenotype from different tissues and organs [5]. To study the biology of EC from a given tissue, the ideal cells should be primary EC from that tissue.

Several methods have been described for the isolation of heart endothelial cells. Perfusion technique has been used to isolate endothelial cells of the heart especially from the coronary artery endothelial cells [6–11]. Magnetic bead cell sorting using single [12] or multiple markers [13–16] has been performed to purify endothelial cells from the heart. Flow cytometry has been used to sort cells after labeling with DiI-Ac-LDL [17, 18]. However, endocytosis of Ac-LDL mediated by scavenger receptors is a specific but not exclusive property of endothelium as macrophage and other vascular cells can uptake Ac-LDL [19]. E-selectin and vascular cell adhesion molecule-1 (VCAM-1) have been used to sort the endothelial cells after the stimulation with tumor necrosis factor-alpha (TNF-*α*). However, the requirement for the pre-stimulation of cells by cytokines prior to sorting can change unique expression of sorted cells [20]. CD31 and Lectin Ulex europaeus I (UEA-I) have been used to sort the endothelial cells from guinea pig heart [21], but endothelial cells from mice do not bind UEA-I [22, 23]. 

In addition, primary EC are needed in clinical applications, which limits the utility of current EC lines. For these reasons, we aimed to isolate and expand a pure population of primary endothelial cells from the murine heart. Herein, we present a new multicolor flow cytometry approach to isolate, characterize, and *ex vivo* expand cardiac endothelial cells. These cardiac EC can be *ex vivo* expanded for more than 15 passages, retained endothelial cell functions *in vitro* and exhibit angiogenic capacity when transplanted *in vivo.*

## 2. Methods 

### 2.1. Animals

C57BL/6J (000664) mice, C57BL/6-Tg (CAG-EGFP) 10sb/J (003291) mice, and B6.129S7-Rag1^tm1Mom^/J (002213) mice were purchased from the Jackson laboratory, Bar Harbor, ME. All experimental procedures were approved by the Institutional Animal Care and Use Committee of the University of Washington.

### 2.2. Cell Harvesting and Processing

Hearts from two mice per group were pooled and treated as an individual sample. Hearts were divided into atrium and ventricle under the microscope (Stereomaster, Fisher Scientific, Waltham, MA) then dissociated into mononuclear cells as previously described with minor modification [24]. Briefly, hearts were minced with dissecting scissors into ≤3 mm pieces and digested with 2 mg/mL Collagenase type IV (Worthington, Lakewood, NJ) and 1.2 units/mL Dispase II (Worthington) and 2 mM CaCl_2_ in PBS at 37°C for 45 minutes with agitation every 15 minutes. After 45 minutes, enzymes were neutralized by adding twice the original volume of Ham's F10 with L-Glutamine (HyClone) and 15% horse serum (HyClone), filtered through sterile 70 *μ*m nylon mesh cell strainer (Fisher Scientific), centrifuged at 300 g for 5 minutes, and resuspended in hemolytic buffer (155 mM NH_4_Cl, 10 mM KHCO_3_ and 0.1 mM EDTA in H_2_O) for 5 minutes at room temperature. Then, cells were resuspended in PBS, counted and either transferred to culture immediately or to 1.7 mL Eppendorf tubes for flow cytometry staining. 

### 2.3. Immunofluorescence

Tissues were embedded in an embedding mold, fill the mold with Optimal Cutting Temperature compound (Tissue-Tek O.C.T., Sakura Finetek, Torrance, CA), rapidly submerge the mold into 2-methylbutane (J. T. Baker, Phillipsburg, NJ) cooled with liquid nitrogen and sectioned (5–10 *μ*m thickness). Slides were washed with Phosphate Buffer Saline (PBS, HyClone, Logan, UT), and endogenous biotin were blocked by streptavidin/biotin blocking kit (Vector, Burlingame, CA) according to the manufacturer's protocol before staining if biotinylated primary antibody is used. Cultured cells from the heart were fixed with 2% paraformaldehyde before staining. 

 All primary and secondary antibodies were diluted with 1% Bovine Serum Albumin (BSA, EMD Chemicals, Gibbstown, NJ) in PBS and then incubated 1 hour at room temperature with tissues or culture cells. Controls with only secondary antibodies were included for all staining. 

The following primary antibody were used: fluorescein isothiocyanate (FITC) antimouse CD31 (1 : 50, clone 390, BD Biosciences, San Jose, CA or eBioscience, San Diego, CA), biotin antimouse Sca-1 (1 : 500, clone D7, BD Biosciences or 1 : 400, eBioscience), purified rat anti mouse CD34 (1 : 50, clone RAM34, BD Biosciences), polyclonal rabbit anti human vWF (1 : 100, Dako, Carpinteria, CA), rabbit polyclonal Ab to eNOS (1 : 100, Abcam, Cambridge, MA), and rabbit polyclonal Ab to Caveolin 1 (1 : 300, Abcam), monoclonal anti-*α* smooth muscle actin Cy3 (1 : 400, clone 1A4, Sigma, St. Louis, MO), rabbit polyclonal Anti-NG2 Chondroitin Sulfate Proteoglycan (1 : 200, Chemicon, Billerica, MA), rabbit polyclonal anti-GFP (1 : 100, Abcam, Cambridge, MA). 

 The following secondary antibodies were used: Avidin-Texas red (1 : 500, Vector), Alexa Fluor 594 chicken antirat IgG (1 : 1000, Invitrogen, Carlbad, CA), Streptavidin-Alexa fluor 594 conjugate (1 : 400, Invitrogen), Alexa 647 goat anti-rabbit IgG (1 : 1000, Invitrogen), Alexa 488 goat anti-rabbit IgG (1 : 1000, Invitrogen), and Alexa 594 goat anti-rabbit IgG (1 : 1000, Invitrogen).

Tissues and cells were also stained with 4′,6-Diamidino-2-phenylindole dihydrochloride (DAPI) to visualize the nuclei and examined by Axiovert 200 fluorescence microscopy (Zeiss, Thornwood, NY). Monochromatic images were acquired with the manufacturer's software and taken with the same parameters and exposure time as negative control. Images for Alexa 647 were taken using gamma settings. Images were assembled in Adobe Photoshop CS2.

### 2.4. Flow Cytometry and Cell Sorting

Hearts from 3-week-old to 30-month-old (*n* = 32) C57BL6/J or C57BL/6-Tg (CAG-EGFP) 10sb/J (*n* = 6) mice were used for flow cytometry analysis. Mononuclear cells dissociated from the murine hearts were incubated with CD45, CD31, CD34, and Sca-1 antibodies, as outlined below in 100–300 *μ*L (10^6^ cells per 100 *μ*L of antibody) of 0.3% BSA in PBS 1 hour on ice in the dark, washed, and analyzed by either BD FACSAria or BD FACSAriaII Flow cytometers. If biotin antimouse CD34 was used, cells were incubated with biotin anti-CD34 for 1 hour then washed and incubated with streptavidin-fluorophore conjugate for 1 hour.

The antibodies used in most experiments were Phycoerythrin (PE) Cy5 antimouse CD45 clone 30-F11 (all eBioscience, unless otherwise indicated), PECy7 antimouse CD31 clone 390, biotin antimouse CD34 clone RAM34, streptavidin-phycoerythrin-TexasRed conjugate (Invitrogen), and allophycocyanin (APC) antimouse Sca-1 clone D7. The antibodies used in some other experiments were PerCP antimouse CD45 clone 30-F11 (BD Biosciences), Alexa Fluor 700 antimouse CD45, FITC antimouse CD31, PE antimouse CD31, PECy7 anti-CD34, Pacific Blue antimouse CD34, streptavidin-Pacific Blue conjugate (Invitrogen), streptavidin-Alexa 647 conjugate (Invitrogen), and eFluor  605^NC^ antimouse Sca-1. The other antibodies also made from the same clone as those in main experiment. 

The concentration of antibodies used was 600 ng per 10^6^ cells except for Sca-1 APC and streptavidin-fluorophore conjugate which was used at 300 ng per 10^6^ cells due to their strong avidity. 10^5^ cells stained with 200 ng of each antibody/fluorophore as well as one tube of 10^5^ unstained cells were used to compensate the overlapping of fluorophores. Forward and side scatter were used to exclude cell debris, doublets and aggregates. Hematopoeitic cells were excluded from the plot on the basis of CD45+ cells. Next, CD45−, Sca-1+, and CD31+ cells were identified as heart endothelial cells and sorted into growth medium for culture and RLT lysis buffer (79216, Qiagen, Valencia, MD) containing *β*-Mercaptoethanol (M7522, Sigma) 10 *μ*L/mL for reverse transcription. Flow cytometry data analysis and graph generation were performed by FlowJo 7.5.4. Gating parameters were adjusted based on the fluorescence histograms for the positive and negative controls. 

### 2.5. Cell Culture and Growth Kinetics

Hearts from six female C57BL/6-Tg (CAG-EGFP) 10sb/J mice (3, 4, 6-month-old) were harvested aseptically. Mononuclear cells both acquired directly from the heart (unsorted cells, US) and sorted by flow cytometer (sorted cells, S) were resuspended in 5 mL growth media and seeded on uncoated 100 × 20 mm of tissue culture dishes (BD bioscience) at 37°C, 5% CO_2_, 5% O_2_ in a humidified incubator. Growth media contained high glucose Dulbecco's Modified Eagle's Medium (DMEM) supplemented with 10% USDA Tested fetal bovine serum, 100 units/mL Penicillin, 100 *μ*g/mL Streptomycin (all from HyClone), 10 ng/mL recombinant mouse VEGF (R&D, Minneapolis, MN) and 20 ng/mL recombinant mouse basic FGF (R&D). Medium was changed every 3-4 days or every passage to a new dish. At each subsequent passage, cells were enumerated for calculation of a growth kinetic curve. The number of cell doubling occurring between passages was calculated according to the equation: Cell doubling = log⁡_2_(*C*_*H*_/*C*_*S*_), where *C*_*H*_ is the number of cells at harvest and *C*_*S*_ is the number of cells seeded. The sum of all previous cell doubling determined the cumulative cell doubling at each passage.

### 2.6. Reverse Transcription Polymerase Chain Reaction and Real-Time Quantitative Polymerase Chain Reaction

Total RNA was prepared using RNeasy Mini kit (Qiagen) and treated with DNase (Qiagen or Promega, Madison, WI) according to the manufacturer's protocol. First strand cDNA was created using 1.2–2 *μ*g of DNase-treated total RNA from cultured cells and 0.1–1 *μ*g of DNase-treated total RNA from freshly sorted cells using the High Capacity cDNA Reverse Transcription Kit (Applied Biosystems, Carlbad, CA) as per the manufacturer's recommendation. RNase inhibitor (Applied Biosystems) was also added in the reverse transcriptase reaction at the concentration of 1 *μ*L per reaction. The cycling parameters were 95°C for 7 min initial activation followed by 94°C for 30 sec, 55°C for 30 sec, and 72°C for 45 sec for 35 cycles, followed by 72°C for 5 min. The PCR reaction was performed using Immomix PCR mastermix (Bioline, Randolph, MA). PCR products were run on 2% Agarose gel at 110 volts for 45–60 min. 

For the quantitative PCR, all reactions were set up using *Power* SYBR Green PCR Master Mix and performed and analyzed using the ABI 7900HT PCR system and SDS 2.2 analysis software (all from Applied Biosystems). The threshold cycle (Ct) value for each gene was normalized to the Ct value of glyceraldehyde 3 phosphate dehydrogenase (GAPDH). The relative mRNA expression was calculated by using the formula; 2^−ΔΔCt^, where ΔCt = Ct_sample_ − Ct_GAPDH_  and  ΔΔCt = ΔCt_sample_ − ΔCt_reference group_. Primers were designed by primer express 3.0 (Applied Biosystems). The oligonucleotide sequences are listed in Table 1.

### 2.7. *In Vitro* Functional Assays: Tube Formation Assay and Uptake of DiI-Acetylated-Low-Density Lipoprotein

Pure growth factor reduced Matrigel matrix (BD Biosciences) was incubated at 37°C at least 3 hours before use [25]. Cultured sorted cardiac endothelial cells were seeded at a density of 13,000, 26,000 and 52,000 cells/cm^2^ and incubated at 37°C, 5% CO_2_, and 5% O_2_. Cells were observed at 1.5 hour, 3 hour, 5.5 hour, and overnight by visual microscopy with inverted microscope at 10x magnifications for capillary-like formation. 

To assess the ability of endothelial cells to incorporate acetylated LDL, attached cells were incubated with 10 *μ*g/mL DiI-Ac-LDL (Biomedical Technologies, Stoughton, MA) in growth media for 3 hours at 37°C, 5% CO_2_ and 5% O_2_ [26]. Cells were washed 3 times and stained with DAPI. Cells were examined by using fluorescence microscopy filter set 43HE for DiI-LDL and filter set 49 for DAPI. 

### 2.8. *In Vivo* Transplantation

Three types of *in vivo* transplantations were conducted. All cells came from CAG-EGFP mice donors. Mice were anesthetized with isoflurane before performing the transplantation. For Matrigel plug assay, 2–5 month-old male C57BL/6 mice were subcutaneously injected with 4 × 10^6^ GFP cultured cells at 67 days in culture from both atrium and ventricle as well as from both unsorted and sorted culture cells (*n* = 3/cell line, Total mice = 12). Cells were diluted in 100 *μ*L of Matrigel and 50 *μ*L of growth media. After 14 days, the mice were injected with Rhodamine labeled wheat germ agglutinin (WGA) 30 minutes before being sacrificed. Matrigel plugs were then removed, embedded in OCT, and cryosectioned. The sections were stained with goat polyclonal Ab to GFP (1 : 100, Abcam) and rabbit polyclonal Ab to Caveolin-1 (1 : 300, Abcam) followed by Alexa 488 donkey antigoat IgG and Alexa 488 goat anti-rabbit IgG (both 1 : 1000, invitrogen) respectively to visualize with fluorescent microscopy. 

 For intravenous (IV) injection via tail vein, atrium FACS sorted cells that have been cultured for 5 passages were labeled with PKH26 (Sigma) which is a lipophilic marker inserted into the membrane of viable cells according to manufacturer's protocol prior to injection for better cell tracking [27]. Then, cells were resuspended in PBS with 0.5% KnockOut Serum replacement (Invitrogen) 100 *μ*L per mouse. 6-month-old female C57BL6 mice (*n* = 2) were injected with 2.5 × 10^6^ cells and sacrificed after 1 day [25], after 4 weeks (*n* = 5), and after 2 months (*n* = 3). Mice harvested after 2 months of transplantation were injected with biotinylated wheat germ agglutinin (0.5 mg/mL, Vector) before harvesting. 

For intra-arterial injection via cardiac ventricle, cells from sorted atrium in culture were trypsinized and labeled with PKH26 before injection. 2.5 × 10^6^ cells were injected transthoracically into ventricle of 7-month-old male (*n* = 1) and 2-month-old female (*n* = 2) Rag1 null mice. Tissues were harvested 2 weeks after transplantation.

### 2.9. Western Blotting

Tissues were snapped frozen in liquid nitrogen and kept in −80°C until homogenization. For homogenization, 500 *μ*L of RIPA buffer (containing 150 mM sodium chloride, 1% Triton X-100, 0.5% sodium deoxycholate, 0.1% sodium dodecyl sulphate, 50 mM Tris, and protease inhibitors) were added to each tissue and then triturated. Then samples were kept on ice and vortexed every 5–10 minutes for 1 hour. Samples were then centrifuged for 20 min at 12000 rpm at 4°C in a microcentrifuge. Then the protein supernatants were transferred to new tubes and kept at −80°C. Approximately 25 *μ*L of the protein lysates were loaded on precast polyacrylamide gels (4–20%) and run by electrophoresis of constant voltage 150 mV for approximately 45 minutes. Gels were transferred to PVDF membranes using semiwet conditions. Complete transfer and visualization of the proteins was assessed by Ponceau Red staining of the membranes. Membranes were blocked with 5% BSA for 1 hour at room temperature. Then membranes were incubated with polyclonal rabbit anti-GFP antibody (Abcam, Cambridge, MA) or eNOS (Ser-1177), phosphor-specific (ECM Biosciences) diluted 1 : 1000 in TBS-T buffer containing 5% BSA for 1 hour, then washed TBS-T buffer for 10 minutes 3–5 times. Membranes were incubated with antirabbit HRP antibody (Vector, Burlingame, CA) diluted 1 : 10,000 in TBS-T buffer containing 5% BSA for 1 hour, then washed TBS-T buffer for 10 minutes 3–5 times. Membranes were incubated with chemiluminescence reagents (ECL, Thermo, Rockford, IL) and visualized using X-ray films. Membranes were stripped and reincubated with antimouse *β*-actin (Abcam) diluted 1 : 40,000 and developed as described above. 

### 2.10. Statistical Analysis

Statistical analysis of the results between groups was performed using the Student *t*-test or ANOVA. Data were presented as the mean ± SD. *P* less than 0.05 was considered to be statistically significant. All tests of significance were 2 tailed.

## 3. Results 

### 3.1. Immunohistochemical Detection of Endothelial Cells in Murine Heart Tissues

**Figure 1:**
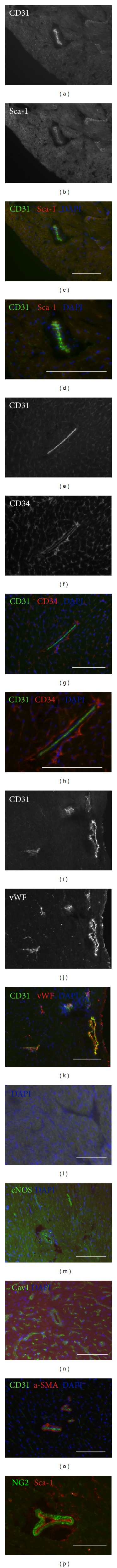
Murine cardiac endothelial cells. Heart tissue sections staining demonstrated (a–d) colocalization of CD31 and Sca-1, (e–h) CD31 and CD34, and (i–k) CD31 and vWF at endothelial lining layer. (l) Representative photograph of a heart section stained with secondary antibodies only (anti rat Alexa 488 and anti rabbit Alexa 594) and DAPI shows acceptable green and red background fluorescence. (m, n) eNOS and Caveolin-1 highlighted the endothelial layer. (o) alpha-SMA exclusively stained the smooth muscle cells of vessels without overlapping with CD31 which stained endothelial cell layer. (p) Staining of NG2 and Sca-1 showed Sca-1 staining in endothelium of coronary arteries and capillaries whereas NG2 is exclusively expressed by vascular smooth muscle cells. Photographs were taken with Zeiss Axiovert 200 using Axiovision v4.6.3 software. Scale bar = 100 *μ*m.

In order to determine the best markers to isolate murine cardiac endothelial cells by flow cytometry, we first study the expression of several endothelial and other cell surface markers by immunohistochemistry of murine heart tissues. Whole heart tissues were stained with CD31, Sca-1, CD34, vWF, eNOS, Caveolin-1, alpha-SMA, and NG2 (Figure 1). CD31, vWF, eNOS, and Caveolin-1 mostly stained endothelial cells while Sca-1 and CD34 stained both endothelial and perivascular cells. Endothelial cells in murine heart can be distinguished from vascular smooth muscle cells by exclusive CD31 staining and lack of alpha-SMA. Also, endothelial cells stained positive for Sca-1 but negative for NG2 which is expressed by pericytes and vascular smooth muscle cells [28].

### 3.2. Cell Sorting by Flow Cytometry for Cardiac Endothelial Cells

**Figure 2:**
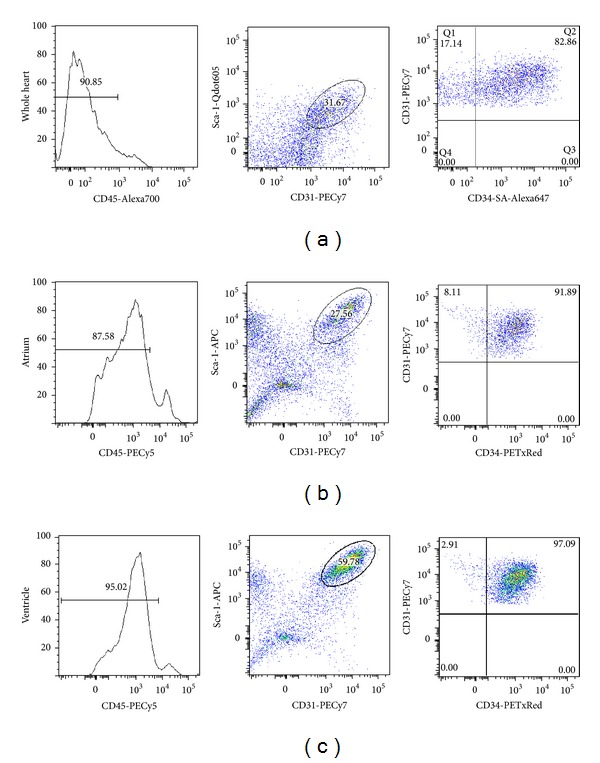
Flow-cytometry analysis of primary isolates of mouse cardiac endothelial cells. After mechanical disaggregation and enzymatic digestion, single cell suspension from murine whole heart (a), atrium (b), and ventricle (c) were incubated with combination of fluorescent-antibody to CD45, Sca-1, CD31, and CD34. Cells were first gated to include only small and low granulated cells from FSC-A and SSC-A dot plot. Hematopoietic cells identified by CD45+ were gated out. Sca-1+/CD31+ were identified as endothelial population. This population was further divided into CD34+ or CD34− endothelial cells.

Based on the immunohistochemistry of the heart using cell surface markers to identify endothelial cells, we designed a flow cytometry protocol to specifically isolate endothelial cells while excluding other major mononuclear cell populations such as cardiomyocytes, perivascular cells, hematopoietic cells, and smooth muscle cells. This approach is a modification of a previous flow cytometry protocol developed in our lab to isolated endothelial cells from the skeletal muscle [24]. Cardiac endothelial cells were identified as Sca-1+ and CD31+ which are expressed by not only endothelial cells from large vessels but also those from capillaries. As the heart is a highly perfused organ, hematopoietic cells which can express CD31 and Sca-1 constitute a significant contribution of the heart preparation. Therefore, we first gated out CD45+ cells to eliminate hematopoietic cells (Figure 2). Subsequently, cardiac endothelial cells were sorted as a Sca-1+ and CD31+ population, and most of this population is CD34+. The same method was applied to the whole heart, atrium, and ventricle. Preparations from segregated atria and ventricles seem purer as the Sca-1+/CD31+ cell population from segregated atrium and ventricle has more CD34+ cells than the whole heart. This indicates that the whole heart preparations contained a population of Sca-1+/CD31+ endothelial cells that are negative for CD34, which could represent cells from the aortic-pulmonary trunk or valves.

**Figure 3:**
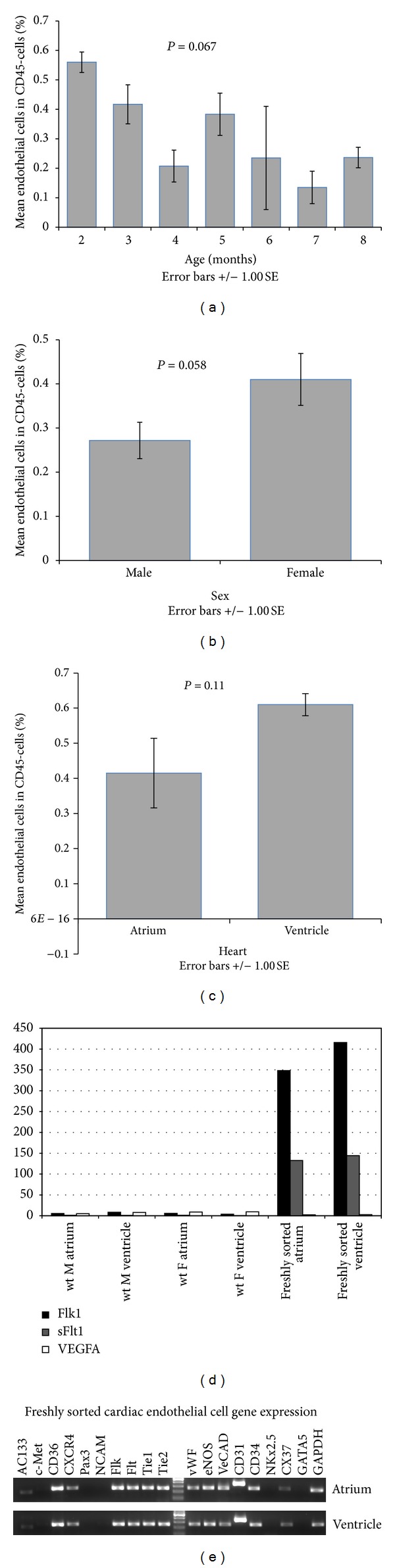
Freshly-sorted cardiac endothelial cells. Comparison of endothelial percentage within CD45− population among age (a) (*n* = 28) and gender (b) (*n* = 29). (c) The percentage of ECs within CD45− population between atrium and ventricle in 3-month-old mice (*n* = 11). (d) q-RT-PCR levels for *Flk-1*, soluble *Flt-1,* and *VEGF-A* of one male (M), one female (F), and freshly FACS-sorted ECs from atrium and ventricle normalized to GAPDH. (e) Representative RT-PCR analysis of freshly FACS-sorted ECs from atrium and ventricle (three females 15–24-month-old) showed expression of endothelial markers Flk-1, Flt-1, Tie-1, Tie-2, vWF, eNOS, VE-Cad, CD31, and CD34 while lacked cardiomyocyte marker NKx2.5 and endocardium marker GATA5. *P* values were calculated by ANOVA (a) or Student's *t*-test (b, c).

Cardiac endothelial cells represent 33.06 ± 3.4% of all CD45− mononuclear cells isolated. There is a tendency of decreased abundance of cardiac endothelial cells with age (*n* = 28 mice from ages 2–8 month-old) but is not statistically significant (Figure 3(a)). Also there is a tendency of less abundance of cardiac endothelial cells in males compared to females (*n* = 29, 16 males and 13 females) (Figure 3(b)). In addition, the percentage of endothelial cells is lower in atrium than in ventricle but is not statistically significant (*n* = 11) (Figure 3(c)). Quantitative RT-PCR for Vascular Endothelial Growth Factor Receptor 2 (Flk-1) [29] and its inhibitor soluble Flt-1 [30] and VEGF-A, normalized to whole heart cDNA, in freshly FACS-sorted cardiac endothelial cells from the atrium and ventricle compared to their respective tissues revealed that Flk-1 and sFlt-1 are highly expressed in the FACS-sorted cardiac EC population (Figure 3(d)) indicating our flow cytometry purification is efficient as these are endothelial specific genes [31]. On the other hand, VEGF expression was not enriched in cardiac EC fraction compared to the whole tissue indicating that cardiac cells other than endothelial cells contribute to VEGF expression [32, 33]. 

RT-PCR of freshly sorted cardiac ECs confirms the expression of many endothelial cell markers such as *Flk-1* (VEGF receptor-2), *Flt-1* [29, 34] (VEGF receptor-1), *Tie-1* and *Tie-2 *[34, 35] (angiopoietin receptors), *vWF* [36–38], *eNOS* [39, 40], *VE-Cadherin* [41], and *CD31* [42, 43]. *CD34 *[44, 45] and *CD36* [46] which are expressed by microvascular ECs are also positive. Conversely, cardiac EC cells are negative for *NKx2.5* and *GATA5* which should only be expressed by cardiomyocyte and endocardial cells, respectively [47–50]. *CXCR4* which is an important chemotaxis receptor [51–53] is positive in cardiac EC, but other markers associated with adhesion and migration such as *c-Met* (mesenchymal-epithelial transition factor), *Pax3* (Paired box gene 3), and *NCAM* (neural cell adhesion molecule) are negative [31, 54–57]. AC133, which biological function is not well understood but is used as a marker for endothelial progenitor cells [58], is also positive in freshly sorted cells. Connexin-37, the gap junction protein [59], is expressed in freshly sorted cells from both atrium and ventricle. The expression profile for all these genes on freshly sorted cells is similar from both atrium and ventricle (Figure 3(e)).

### 3.3. Optimization of Culture Conditions

Initially, we grew cardiac ECs in high glucose DMEM supplemented with 10% USDA tested fetal bovine serum, 10 ng/mL recombinant mouse VEGF at 37°C, 5% CO_2_, 20% O_2_. Under these conditions, FACS-sorted EC did not grow and quickly underwent cell death within the first week of culture. Then, we cultured the whole heart mononuclear cells in physiological hypoxia [60], 5% O_2_. Cells grew for a few passages but then subsequently died. We hypothesized that supplementation with basic fibroblast growth factor (bFGF) in addition to VEGF may help the growth and maintenance of cardiac endothelial cells as bFGF is pivotal for heart development and vascularization [61, 62]. Following supplementation of the media with 20 ng/mL recombinant mouse basic FGF, we could maintain cardiac endothelial cells for long-term culture. All the experiments described below are from cardiac endothelial cells derived from transgenic mice expressing EGFP driven by the chicken *β*-actin promoter (CAG-EGFP) mice *ex vivo* expanded in the conditions described herein. 

### 3.4. Endothelial Phenotype and Functional Capabilities of Cultured Cells

**Figure 4:**
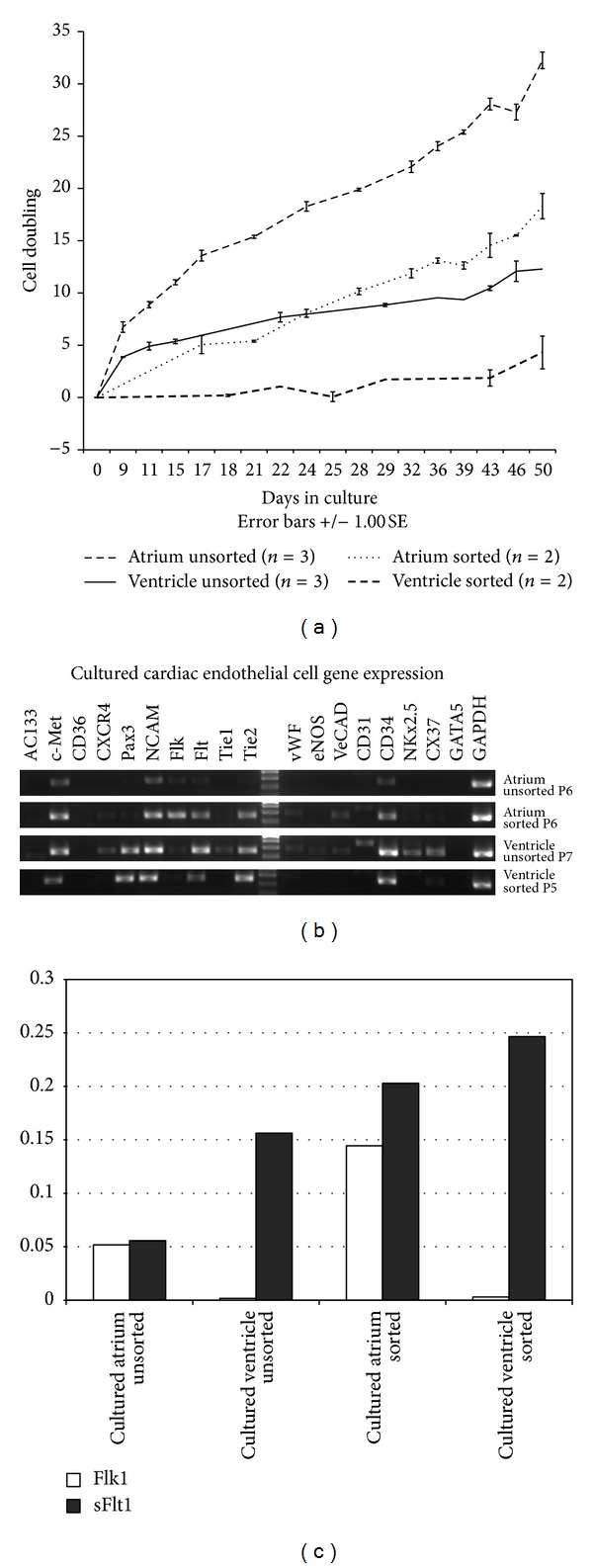
Cardiac endothelial cells in culture. (a) growth kinetics comparison of cardiac endothelial cells between unsorted and FACS-sorted cells derived from six female mice. (two 3-month-old, two 4-month-old and two 6-month-old). Each age group was combined into one sample. Cardiac endothelial cells were culture in DMEM with 10% FCS, 10 ng/mL VEGF, and 20 ng/mL bFGF for 50 days in 5% CO_2_ and 5% O_2_ incubator. (b) Representative RT-PCR analysis of cultured ECs at specified passages from atrium and ventricle. (c) q-RT-PCR for *Flk-1* and soluble *Flt-1* in cultured ECs from the same specimens as (b) normalized to GAPDH.

We compared the proliferation kinetics of endothelial cells from atrium unsorted, atrium sorted, ventricle unsorted, and ventricle sorted cells. Remarkably, the unsorted cells from both atrium and ventricle proliferated faster than the sorted ones. However, the unsorted cells contained a heterogeneous population of cells which may grow faster than pure population of endothelial cells. Although the % of EC from atrium is lower than in ventricle, cells from atrium grew faster than those from ventricle (Figure 4(a)). All cultures from atrium successfully grew in culture whereas one cell line from ventricle sorted source did not grow beyond the first passage, indicating ventricle cells are intrinsically harder to grow under these conditions.

Analysis of RT-PCR of cultured cells after passage 6-7 revealed downregulation of *AC133, CD36,* and *CXCR4* whereas *c-Met, Pax3* (mostly in ventricle), and *NCAM *were upregulated as compared to freshly FACS-sorted cells (Figure 4(b) versus Figure 3(e)). Expression of many endothelial cell genes were downregulated in culture especially *Tie-1* and* eNOS*. The comparison of unsorted and sorted cultured populations revealed enrichment of endothelial cell markers in FACS-sorted atrium EC compared to unsorted atrium cells such as *Flk-1, Flt-1, Tie-2, vWF, VE-Cad,* and *CD31*. These results indicate that the atrium sorted cells are more homogenous than the atrium unsorted cells. Likewise, comparison of ventricle FACS-sorted endothelial cells and unsorted cells showed expression *NKx2.5* in ventricle unsorted cells which indicate contamination of cardiomyocyte-like cells in the unsorted population. *Connexin 37* expression was only maintained in ventricle unsorted cells (Figure 4(b)). Interestingly, *Flk-1* expression was maintained in cultures from atrium but seemed decreased in cultures from ventricle compared to freshly FACS-sorted EC (Figure 4(b) versus Figure 3(e)).

We hypothesized that differences in *Flk-1* expression by cultured cells may explain why ventricle cells grew slower than cells from atrium. Quantitative RT-PCR for *Flk-1* and *sFlt-1* confirmed the RT-PCR results (Figure 4(c)). Higher level of *Flk-1* in cultured cells from atrium may explain why cells from atrium (both unsorted and sorted) grow faster than cells from ventricle as we supplement with VEGF in culture and only cells that express *Flk-1* will respond to VEGF mitogenic activity [64]. Moreover, the *sFlt-1* which is an angiogenic inhibitor [65] is highest in cultured FACS-sorted EC from ventricle. Thus, endothelial cells from atrium may be better than ventricle EC for *ex vivo* expansion under these conditions.

**Figure 5:**
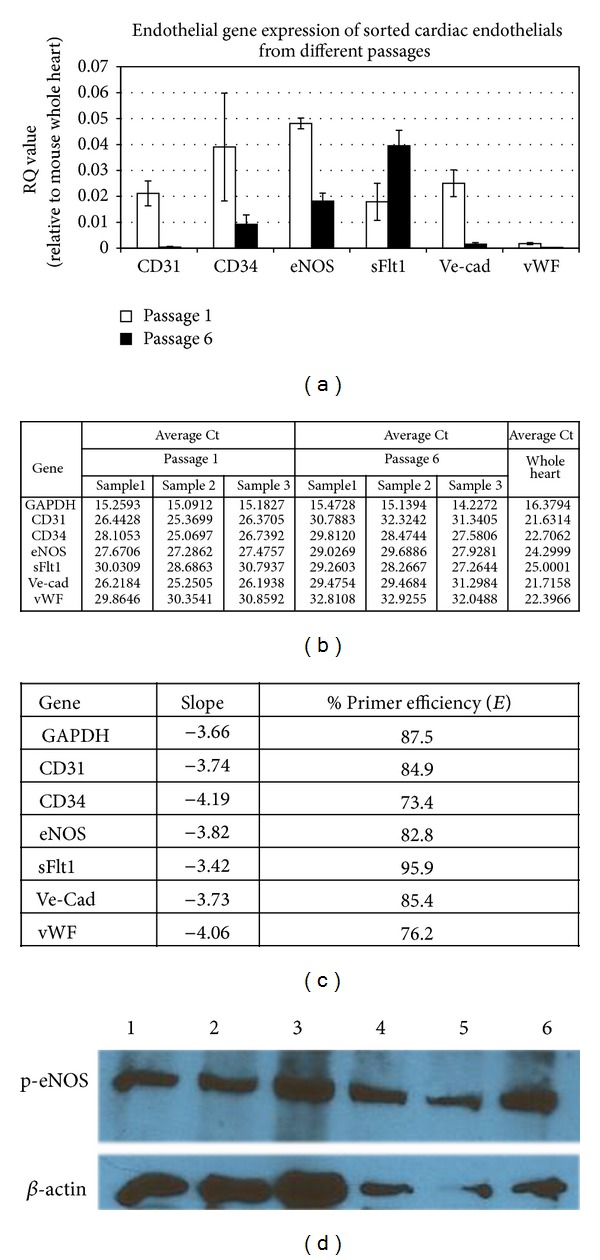
Endothelial marker expression in early and late cultured cardiac endothelial cells. (a) q-RT-PCR of endothelial cell genes revealed a decreased expression of *CD31, CD34, eNOS, VE-Cad,* and *vWF* in late cultured EC (passage 6, black bar) compared to early cultured EC (passage 1, white bar). In contrast, expression of *sFlt-1* was increased. (b) the table shows average Ct values of each endothelial genes expressed by sorted cardiac endothelial cells from atrium in passage 1 and 6. The experiment was run from three different samples in each passage represented as Sample 1, 2, and 3, respectively. Each sample was performed in triplicate per each target gene. The RNA from mouse whole heart was used as positive control. (c) the table represents efficiency (*E*) of primers that we used for q-RT-PCR. We calculated the percentage of primer efficiency by using the formula; *E* = [10^∧^(−1/slope)] − 1 × 100 where *E* is the primer efficiency of the q-RT-PCR reaction and slope refers to the slope of the plot of Ct value versus the log of the input RNA amount. Our primer list demonstrates a slope between −4.19 and −3.42 which corresponds to an estimated efficiency between 73.4% and 95.9%, respectively [63]. (d) western blot for phosphorylated eNOS showed active eNOS in both early (passage 1 samples 1, 2, and 3) and late passaged EC (passage 6 samples 4, 5, and 6).

 To confirm the RT-PCR results we performed q-RT-PCR for the genes whose expression change from early compared to late passage. Although the expression levels of *CD31, CD34, eNOS, VE-Cad,* and *vWF *were downregulated in late passage, these genes were still detectable (Figures 5(a)–5(c)). We then confirmed the protein expression of active (phosphorylated) eNOS in both early and late passage cultured ECs (Figure 5(d)). This indicates that although late passaged EC downregulate the expression of some EC genes, they still produce and activate proteins important for their function, such as eNOS.

**Figure 6:**
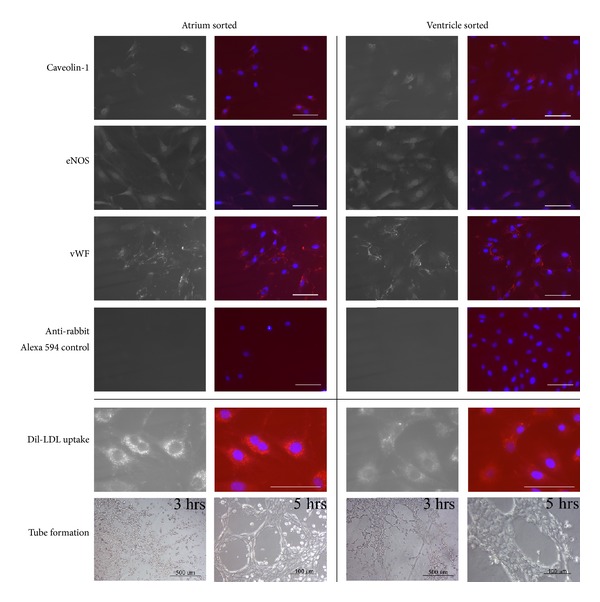
Immunophenotype and functional analysis of cultured FACS sorted cardiac endothelial cells. The upper panel were representative photograph of immunofluorescent staining of cultured FACS sorted cardiac endothelial cells for antiCaveolin-1, anti-eNOS, and anti-vWF followed by anti rabbit IgG Alexa 594. Staining with only secondary anti rabbit IgG Alexa 594 antibody shows minimal background red fluorescence. Left panel shows the monochromic photograph for each antibody. Right panel shows each antibody in red and DAPI in blue. Scale bar = 100 *μ*m. The lower panel were cultured FACS sorted cardiac endothelial cells up took Ac-LDL-rhodamine (red), also stained with DAPI (blue) and cultured FACS sorted cardiac endothelial cells were plated in Matrigel for the formation of capillary-like structures. Photographs were taken at 3 hours (scale bar = 500 *μ*m) and 5.5 hours (scale bar = 100 *μ*m) after incubation.

Sorted cardiac ECs maintain their phenotype and functional capabilities *in vitro*. After long-term culture, FACS-sorted endothelial cells from atrium (passage 22) and ventricle (passage 19) were still positive for endothelial cell markers such as Caveolin-1, vWF, and eNOS by immunohistochemistry (Figure 6). Cells were also capable of acetylated-LDL uptake [66]. Cultured cells also formed vascular tubes when cultured on pure matrigel [67]. However, atrium sorted cells required higher cell density to form vascular tube. Atrium sorted cells can form vascular tubes at minimum density of 52,000 cells/cm^2^, but ventricle sorted cells can form vascular tube at densities of 13,000, 26,000, and 52,000 cells/cm^2^ (data not shown).

### 3.5. *In Vivo* Transplantation of Cultured Cardiac Endothelial Cells

**Figure 7:**
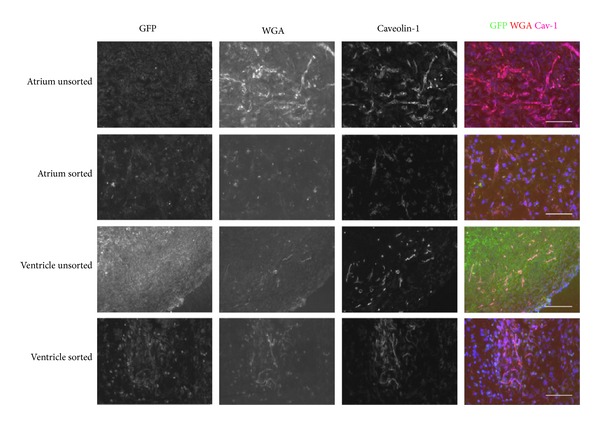
Microvessels in matrigel injected subcutaneously over the dorsum. Unsorted and FACS sorted GFP positive endothelial cells from the heart in matrigel were injected subcutaneously over the dorsum of C57BL6 mice (*n* = 3 for each cell line, total *n* = 12). After 14 days, 0.5 mg/mL WGA-rhodamine (red) was injected intravenously 30 minutes before harvesting the matrigel plug. Tissue sections of matrigel were stained with antiCaveolin-1 followed by a secondary Alexa 647 (magenta pseudocolored) and also stained with DAPI (blue). Scale bar = 100 *μ*m.

The angiogenic capability of the cardiac ECs was tested first with the matrigel plug technique [68]. Matrigel plugs were analyzed 14 after transplantation. WGA-rhodamine, which is a lectin that binds vascular endothelium in many species [69], was injected intravenously in live mice before harvesting the tissues. Upon histological and immunostaining analysis of matrigel plugs, GFP+ cardiac ECs from all cell lines formed vessels that stained positive for Caveolin-1. Some WGA positive cells contained with GFP and Caveolin-1 indicating that donor cells on matrigel plugs connected with the circulatory system. Cells from unsorted atrium seem to have more angiogenic capability than ventricular cells (Figure 7).

**Figure 8:**
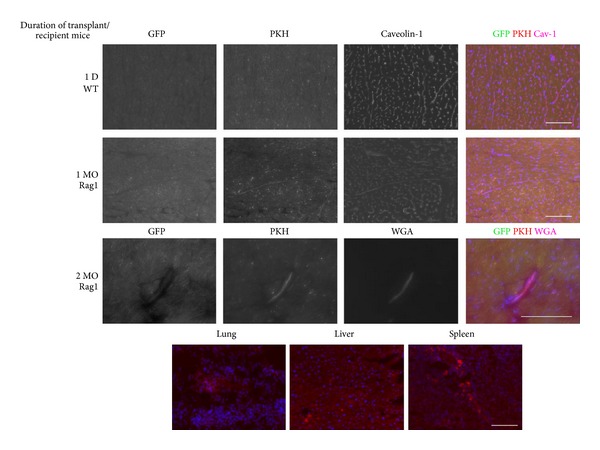
Tissue sections of the heart following cardiac endothelial cells intravenous injection. FACS sorted GFP positive EC from atrium were stained with PKH26 (red) before intravenous injection in C57BL6 mice or Rag1 null mice. Heart tissues were harvested at (A) 1 day after injection in C57BL6 mice (*n* = 2), 1 month (*n* = 5), and 2 months (*n* = 3) in Rag1 null mice. Heart tissue sections were stained with antiCaveolin-1 followed by secondary Alexa 647 (pink) to visualize endothelial cells from 1 day post injected C57BL6 mice and 1 month post injected Rag1 null mice. In 2 month post injected Rag1 null mice, 0.5 mg/mL biotinylated-WGA was injected intravenously 30 minutes before harvesting heart tissue. Then, heart tissue sections were stained with streptavidin-Alexa 647 (magenta pseudocolor) and also stained with DAPI (blue). Bottom: although PKH-26+ GFP+ cardiac endothelial cells were detected in the heart (top), many donor cells (PKH-26+) were found in the lungs, livers, and spleens 4 weeks after transplant. Scale bar = 100 *μ*m.

Since we observed quenching or decay of green fluorescent signal derived from donor cells in the matrigel plug experiments, we labeled cells with PKH26 [27] for better cell tracking prior to transplantation in subsequent experiments. The matrigel plug experiments proved that cardiac ECs formed microvessels and collaterals with existing vessels; thus, we further explored the potential of these cells to home back to their tissue of origin following intravenous injection in the tail vein. After 1 day of intravenous cell injection into wild type mice, some transplanted PKH+ atrium sorted cardiac ECs were found in the heart and costained with Caveolin-1, indicating they were located in vessels (Figure 8). Then, we transplanted PKH+ atrium sorted cardiac ECs into immune compromised Rag1 null mice for longer transplantations. At 1 and 2 months posttransplant, we found PKH+ donor cells in the heart of recipient mice that expressed Caveolin-1 and bound WGA indicating donor EC have integrated in the heart microcirculation (Figure 8).

**Figure 9:**
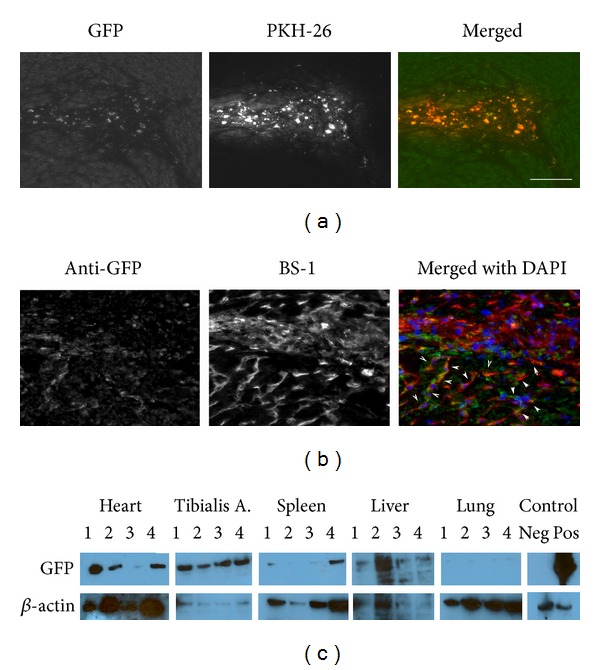
Intra-arterially transplanted cardiac endothelial cells engraft in the heart and other highly perfused tissues. GFP cardiac endothelial cells were stained with PKH-26 and transplanted intra-arterially via transthoracic injections. (a) PKH-26+ GFP+ cells can be found highly concentrated along the injection tract in the left heart ventricle. Note that PKH-26 fluorescence is brighter than GFP and thus this dual labeling method ensures more efficient screening for the localization of the donor cells in multiple tissues. (b) anti-GFP staining identifies donor cells in the injection tract and also migrating out of the tract forming vessels that are BS-1 lectin positive (pseudocolor in red). Arrows highlights the colocalization of GFP+ cells that are also BS1+ (yellow) migrating out of the injection tract. Scale bar represents 100 *μ*m for both (a) and (b). (c) proteins from multiple tissues of four transplanted mice (1, 2, 3, 4) were processed and analyzed by western blotting for GFP. Positive control (heart from transgenic CAG-EGFP mice) shows a predominant band at 54 kDa which correspond to the GFP dimmer. Negative control is untransplanted heart.

However, when we transplanted EC via IV injections, many PKH+ cardiac ECs were trapped in other tissues including lung, liver, and spleen (Figure 8, bottom). Thus, in order to deliver cells directly to the heart, we used intra-arterial injections of 2.5 × 10^6^ cells via transthoracic approach. Fourteen days after transplantation, we found PKH+ GFP+ atrium sorted cardiac ECs along the injection tract in the heart ventricle (Figures 9(a) and 9(b)). Surprisingly, no fibrosis was observed at the injection site. On the contrary, we observed robust angiogenesis in the transplanted site demonstrated by multiple vessels containing PKH+ GFP+ cells that stained positive for the BS1− lectin, a tetrameric agglutinin isolated from *Bandeiraea simplicifolia*, which specifically binds lectin receptors on vascular endothelial cells [69] (Figure 9(b)). Using western blotting for GFP, we confirmed the engraftment of the donor GFP+ cardiac cells not only in the hearts but also in distant organs such as the Tibialis Anterior muscles and less in spleens, livers but almost undetectable in lungs (Figure 9(c)). This indicates that cardiac endothelial cells transplanted via transthoracic injection circulate arterially and engraft in highly perfused tissues but do not get sequestered in the microvenular circulation of the lungs.

**Figure 10:**
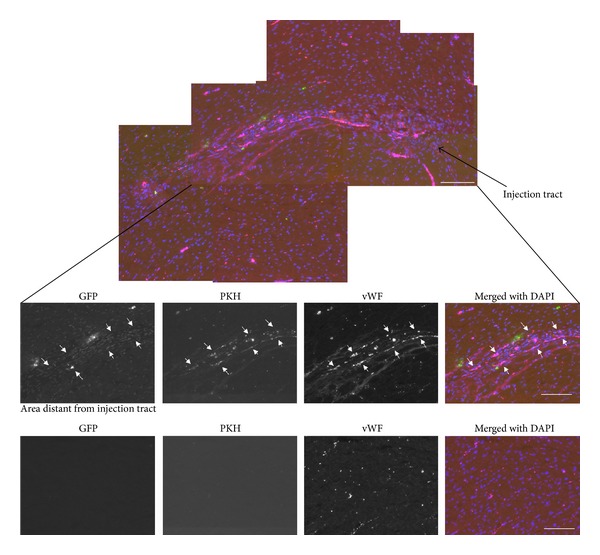
Heart tissue sections after injected cardiac endothelial cells intraarterially. FACS sorted GFP positive endothelial cells from atrium were stained with PKH26 (red) and injected intra-arterially via transthoracic injections in the left ventricle into Rag1 null mice (*n* = 3). Heart tissues were harvested 14 days after injection and were stained with anti von Willebrand factor followed by secondary Alexa 647 (pseudocolored as magenta). Multifluorescence montage of the injection site revealed transplanted cells exhibited robust angiogenesis as they form numerous vWF+ vessels compared to myocardium distant from the injection site (bottom) in which GFP+/PKH+ cells were not detected. Montage is composed by the following fluorescent monochromatic photos: GFP (green), PKH (red), anti-vWF staining (pseudo color as magenta), and DAPI (blue). Scale bar = 100 *μ*m.

A montage of the injected hearts clearly shows enrichment of new vessels in the injection tract (Figure 10). In summary, our results indicate that cardiac endothelial cells transplanted via intra-arterial injection preferentially engraft in the hearts exhibiting robust angiogenesis.

## 4. Discussion

 One of the primary objectives of this study was to establish a protocol that facilitates the identification and selection of microvascular endothelial cells from the heart with a high degree of specificity. Although ECs display many common features, they also exhibit remarkable morphological and functional heterogeneity [70]. Hematopoietic stem cells have been identified prospectively based on their cell surface marker expression, isolated by FACS and transplanted *in vivo*. Flow cytometry and cell surface markers have been instrumental to study the hematopoietic lineage hierarchy and their relationship with other multipotent progenitor populations [71]. We have modified a method previously developed in our lab to isolate pure populations of ECs from skeletal muscle tissue [24] in order to purify and study cardiac endothelial cells. 

The immunofluorescence of heart tissue confirmed that endothelial cells expressed Sca-1, CD31, and CD34 similar to skeletal muscle EC. Therefore, we used Sca-1+/CD31+ to isolate endothelial cells from the heart. Sca-1, a member of Ly-6 antigen family, express in hematopoietic stem cells and a mixture of stem, progenitor and differentiated cell types in a wide variety of tissues and organs [72] including endothelial cells [73]. CD31 is expressed by ECs in multiple tissues and has been used to isolate ECs from the heart of mice [13, 15] and guinea pigs [21]. However, CD31 is also expressed by hematopoietic and immune cells [74]. Since many of these markers are also expressed in hematopoietic cells, we used CD45 to exclude hematopoietic cells that may express Sca-1 and CD31. Most Sca-1+/CD31+ are also CD34+. Using FACS, the percentage of cardiac endothelial cells was not statistically different among various ages, gender, and heart chamber. Enrichment and purity of freshly-FACS sorted cardiac EC were confirmed by high levels of Flk-1 and sFlt-1 expression in FACS sorted fractions compared to whole heart tissue. Interestingly, anatomic dissection and segregation of atriums and ventricles result in different cell recovery and cell growth in culture as compared to whole heart extracts.

Notably, cells from atrium proliferated faster and longer than cells from ventricle. The comparison of endothelial related genes in cultured cells revealed that Flk-1 expression is maintained in cultures from atrium whereas cultures from ventricle expressed low to almost undetectable levels of Flk-1. This may explain why atrium ECs can grow better than ventricle ECs even though atrium has a smaller percentage of ECs than ventricle in fresh heart preparations. Additionally, sFlt-1 is higher in ECs from ventricle, which may inhibit their growth. In addition, vWF and VE-Cad were maintained in cultured atrium ECs whereas cultured ventricle ECs expressed lower levels of these mature endothelial markers. 

Nonetheless, ECs from both atrium and ventricle maintain their phenotype and function *in vitro*. ECs from ventricle required lower cell density to form vascular tubes as compared to atrium EC. We further study the angiogenic capacity of these cultured cells *in vivo*. Cardiac ECs formed vessels and engrafted into existent vessels when transplanted *in vivo* using the matrigel plug technique, intravenous, and intra-arterial injections. Importantly, intravenous injections of atrium sorted cardiac endothelial cells result in their sequestration in the pulmonary microvascular circulation whereas intra-arterially transplanted atrium sorted cardiac endothelial cells were found in the heart and highly perfused organs but barely detectable in the lungs. Thus, to prevent pulmonary embolism, intra-arterial transplantation of cardiac endothelial cells is a more efficient and safer route to deliver cardiac endothelial cells. No tumor or hemangioma was detected in any of these transplants.

This method is highly efficient and specific for the identification, study, and isolation of cardiac ECs. Since we can analyze cardiac endothelial cells directly from freshly dissected heart tissue, this method can serve as a valuable tool to study various heart EC functions and mechanisms in physiology and pathology. Although, the lack of human orthologue of Sca-1 [72] may limit this application, there are many other human endothelial markers such as CD31 that can be applied to develop a similar method in human tissues.

In conclusion, we have developed a highly reproducible method to isolate endothelial cells from murine heart using cell surface markers Sca-1+, CD31+ and CD45−. This method allows direct analysis of cardiac EC for physiological and pathological studies. Furthermore, the angiogenic and homing capacity of cardiac EC transplanted IV and IA makes them ideal candidates for revascularization and neoangiogenic therapies following ischemia.

## Figures and Tables

**Table 1:** Primer sequences for RT-PCR and q-RT-PCR.

Target gene	Forward primer (5′ → 3′)	Reverse primer (5′ → 3′)
*RT-PCR primer sequences *		
AC133	CCATGCAGGAGGAAGTGCTT	TGCTCCACTACATAGTCAATTTGCT
c-Met	TTATTCATGGGCCGGCTTAA	TGGTGATCTTCTTTCCTGTGACA
CD36	CATCCAAATGAAGATGAGCATAGG	CCATGCCAAGGAGCTTGATT
CXCR-4	GCCATGGAACCGATCAGTGT	ACAGGTGCAGCCGGTACTTG
Pax3	CGCTGTCTGTGATCGGAACA	TCTGCTCCTGCGCTGCTT
NCAM	TGCTCGTGTGTCCTCCTTGA	GCTTGGCAGCAACTGACCAT
Flk-1	AAAGACAACGAGACCCTGGTAGAA	CAATGACAAGAAGGAGCCAGAA
Tie-1	TGTTCGTGGCCTCAATGCTA	TCGGATACACACCAAGGCTAAA
Tie-2	GGAACCTGACCTCGGTGCTA	CCCTGAACCTTATACCGGATGA
vWF	GATGTCCAGCTCCCCTTCCT	AGGCGTTTCCGAAGTCTACCA
Flt-1	GGTGAAGATTTGCGACTTTGG	CCGCATGCCTTCCTTCAG
CD34	CTTCTGCTCCGAGTGCCATT	GCCAAGACCATCAGCAAACAC
CD31	GGACTCACGCTGGTGCTCTAT	TTGGATACGCCATGCACCTT
CX43	GAACTCTCCTTTTCCTTTGACTTCA	GTTACAGCGAAAGGCAGACTGTT
CX37	CAGCACACCCACCCTGATCT	GCCTGCCTCCAGCACACT
Nfatc	TCTGGAGAGTCCGAGAATCGA	GTATGGGTAGGAGTAGTTGGACTCGTA
GATA4	CCCGGGCTGTCATCTCACTA	GTGCCCCAGCCTTTTACTTTG
GATA6	AGCAGGACCCTTCGAAACG	GCGCTTCTGTGGCTTGATG
eNOS	TGATGGCGAAGCGTGTGA	GGGCCTGACATTTCCATGAG
VeCad	CCAGCCCTACGAACCTAAAGTG	CACCCCGTTGTCTGAGATGA
NKx2.5	CCAAGTGCTCTCCTGCTTTCC	AGGTACCGCTGTTGCTTGAAG
GAPDH	GGGAAGCCCATCACCATCT	GCCTCACCCCATTTGATGTT
*q-RT-PCR primer sequences *		
Flk-1	ACTGCAGTGATTGCCATGTTCT	CCTTCATTGGCCCGCTTAA
VEGFA	GAGCAGAAGTCCCATGAAGTGAT	CAATCGGACGGCAGTAGCTT
sFlt-1	CAGTTGTCTCTTATCATCTCAGTTTATTGTT	TTTGGGAGGAGCGTTTCCT
CD31	TGCAGGCATCGGCAAAG	GCATTTCGCACACCTGGAT
CD34	CTTCTGCTCCGAGTGCCATT	GCCAAGACCATCAGCAAACAC
eNOS	TTGTCTGCGGCGATGTCA	GAATTCTCTGCACGGTTTGCA
Ve-cad	AGCGCAGCATCGGGTACT	TCGGAAGAATTGGCCTCTGT
vWF	GATGTCCAGCTCCCCTTCCT	AGGCGTTTCCGAAGTCTACCA
GAPDH	GGGAAGCCCATCACCATCT	GCCTCACCCCATTTGATGTT

## Abbreviations

Ab: Antibody
EC(s): Endothelial cell(s)
CD: Cluster of differentiation
Sca-1: Stem cell antigen-1
vWF: von Willebrand factor
eNOS: endothelial nitric oxide synthase
Flk-1: fetal liver kinase 1
Flt-1: fms-like tyrosine kinase 1
Tie-1: tyrosine kinase with immunoglobulin-like and EGF-like domains 1
Tie-2: endothelium-specific receptor tyrosine kinase 2
VE-Cad: Cadherin 5
SV40: Simian Vacuolating Virus
DiI-Ac-LDL: Acetylated Low Density Lipoprotein, labeled with 1,1′-dioctadecyl-3,3,3′,3′-tetramethyl-indocarbocyanine perchlorate
UEA-I: Lectin Ulex europaeus I
US: Unsorted
S: Sorted
BSA: Bovine serum albumin
PBS: Phosphate buffer saline.
